# Intergenerational Technology Programs in Higher Education: From Research to Practice

**DOI:** 10.1093/geroni/igaf122.1378

**Published:** 2025-12-31

**Authors:** Jill Juris

**Affiliations:** Appalachian State University, Boone, North Carolina, United States

## Abstract

This presentation will guide attendees through the presenter’s early career journey of securing funding and developing a practical tool- the InterGenerational Technology Engagement Checklist for Higher Education (IGTECH). Designed for early career scholars and professionals, this session introduces an innovative approach to creating an intergenerational technology program in higher education. Emerging research highlights the benefits of intergenerational technology programs, which bring together multiple generations for collaborative technology learning. Intergenerational technology programs have been shown to reduce social isolation among older adults while also addressing their technology needs. At the same time, younger participants gain professional skills and develop more positive attitudes toward aging. Developed through an RRF-funded multi-stage qualitative study, the IGTECH serves as a guide for university representatives to establish programs that foster meaningful intergenerational relationships through shared technology learning experiences.

